# Pulmonary Langerhans cell histiocytosis with thyroid involvement manifesting as recurrent bilateral pneumothorax and tension bullae in a 3-year-old child

**DOI:** 10.1016/j.ijscr.2019.06.027

**Published:** 2019-06-20

**Authors:** Satoshi Yokoyama, Tatsuo Nakaoka, Daisuke Fukao, Koji Yokoyama, Shigeto Hara, Keigo Hamahata, Akira Yoshida

**Affiliations:** a Department of Pediatric Surgery, Wakayama Medical Center, 4-20 Komatsubara-dori, Wakayama, Wakayama, 640-8558, Japan; b Department of Pediatrics, Japanese Red Cross Society, Wakayama Medical Center, 4-20 Komatsubara-dori, Wakayama, Wakayama, 640-8558, Japan

**Keywords:** LCH, Langerhans cell histiocytosis, PLCH, pulmonary Langerhans cell histiocytosis, PTX, pneumothorax, IPPV, intermittent positive-pressure ventilation, Pulmonary Langerhans cell histiocytosis, Pneumothorax, Tension bullae, Positive pressure ventilation

## Abstract

**Introduction:**

Pulmonary Langerhans cell histiocytosis (PLCH) is a well known entity in adults but is exceedingly rare in children. The main complication of PLCH is the occurrence of pneumothorax (PTX) and tension bullae with subsequent recurrence and persistence despite conservative management.

**Case presentation:**

A 3-year-old child was diagnosed with PLCH with thyroid involvement. Chemotherapy was immediately initiated and continued with chest tube drainage for repeated bilateral PTX. Tension bullae developed in the right lung 1 month after initiating treatment. Emergency resection of the bullae and ligation of pulmonary cysts were performed. On the tenth postoperative day, she developed a tension bulla in the left lung. Emergency thoracotomy was performed. On the tenth postoperative day, chest X-ray and CT suggested return of the right bulla and mediastinal shift, and reoperation was performed. Repeated lung biopsy showed no sign of active LCH. Although her condition improved after the operation, subsequent repeated PTXs occurred and were difficult to treat. She was discharged home after four months. Currently, she is receiving maintenance therapy as an outpatient. During the 12-month follow-up, the pulmonary lesions also showed a tendency for improvement.

**Discussion:**

Although it is difficult to treat recurrent PTX and tension bullae in advanced PLCH, continuous treatment of the primary disease (LCH) and the complications of pulmonary lesions can improve prognosis.

**Conclusion:**

Treatment of PLCH accompanied by recurrent PTX and tension bullae is challenging. The condition can be resolved by treating the primary disease in parallel with combined modality treatment, including surgical operation for complications.

## Highlights

• The main complication of PLCH is the occurrence of pneumothorax (PTX) and tension bullae with subsequent recurrence and persistence despite conservative management.
• Although it is difficult to treat recurrent PTX and tension bullae in advanced PLCH, continuous treatment of the primary disease (LCH) and the complications of pulmonary lesions can improve prognosis.
• Positive-pressure ventilation should be performed with extreme caution in suspected PLCH.

## 1 Introduction

Langerhans cell histiocytosis (LCH) is an uncommon disorder in which S-100- and CD1a-positive cells infiltrate one or more organs, primarily the skeletal system, skin, thyroid gland, liver, lung, spleen and/or hematopoietic system [1]. The lungs are affected in up to 50% of children with multisystem disease, which commonly reflects the total disease activity. The main complication of PLCH is the occurrence of pneumothorax (PTX) and tension bullae with subsequent recurrence and persistence despite conservative management [2,3]. Moreover, involvement of the thyroid gland by LCH is rare, and is usually part of a multisystemic disease in children [[4], [5], [6]]. Here, we report PLCH with thyroid involvement manifesting as recurrent bilateral PTX and tension bullae in a 3-year-old child. This report has been written in concordance with the SCARE criteria Agha et al. (2018) [7].

## 2 Presentation of case

**Fig. 1:**
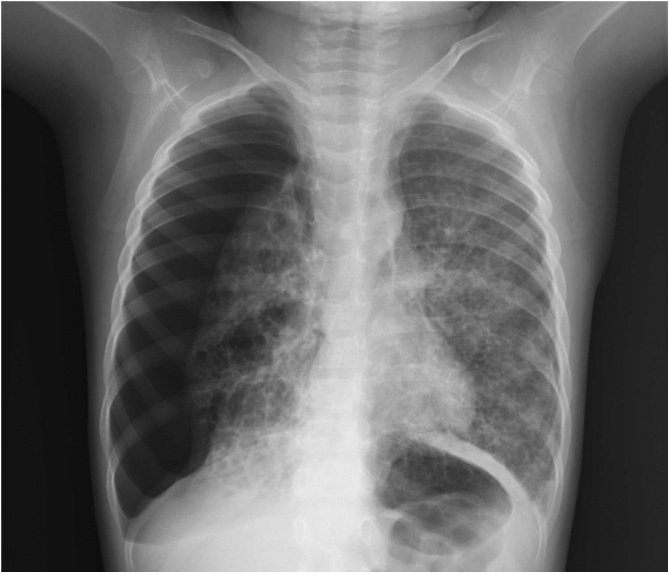
Chest X-ray after chest drainage revealed a right PTX and bilateral bullous lesions at the lung apex.

**Fig. 2:**
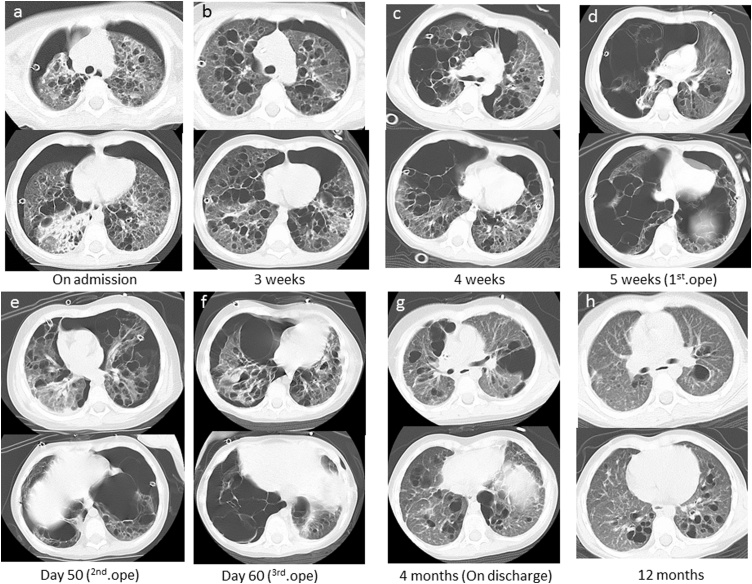
Series of patient’s chest CT.

**Fig. 3:**
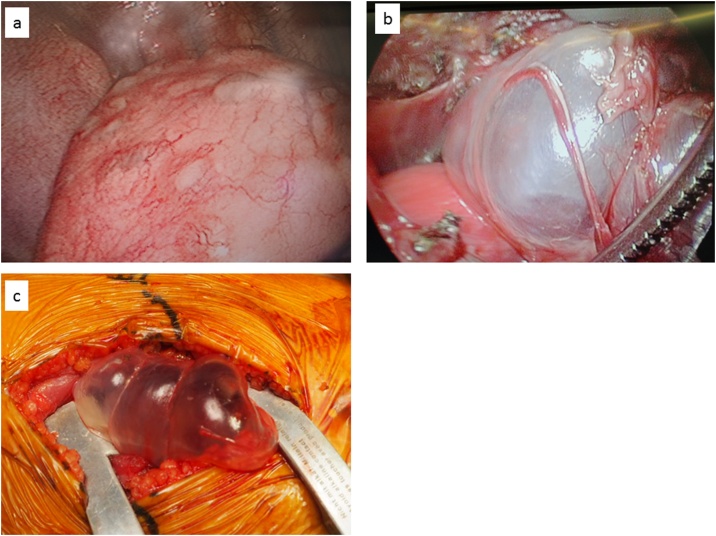
**a**: The thoracoscopic finding shows multiple subpleural cysts on the entire surface of the right lung. **b**: Apperarance of right lung LCH at emergency surgery. **c**: Apperarance of left lung at emergency thoracotomy.

**Fig. 4:**
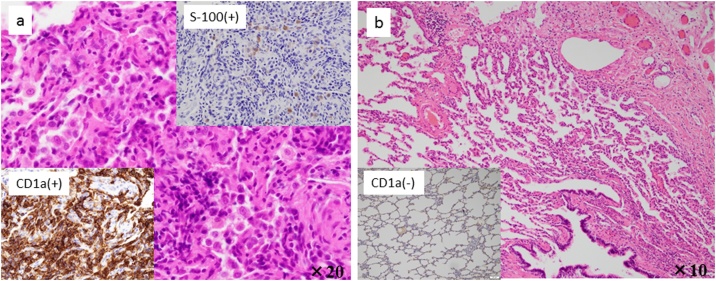
**a**: Lung (Hematoxylin-eosin stain, X 20) shows a proliferation of Langerhans cells, which show elongnated kidney-shaped nuclei. Immunohistochemistry revealed CD1a and S-100 positivity. **b**: Repeated lung biopsy specimen showing CD1a negativity (X 10), suggestive of no active LCH.

A female aged 2 years and 11 months who had been healthy since birth developed sudden dyspnea. She had no relevant medical or family history related to her symptoms. Chest X-ray revealed a right PTX and bilateral bullous lesions at the lung apex (Fig. 1). Chest computed tomography (CT) taken after chest drainage showed bilateral PTX and bilateral cystic changes in the lung parenchyma, predominantly in the upper lobes (Fig. 2a). Contrast-enhanced CT also demonstrated a diffusely enlarged thyroid gland with a spotted low-density area. Laboratory investigations revealed elevated thyroid-stimulating hormone (TSH) of 20.17μIU/ml with low T3 and T4 levels. Thoracoscopy was performed because of the histological diagnosis of this uncommon cystic lung and thyroid gland biopsy was performed. Under thoracoscopic view, multiple bullous lesions were found to occupy most of the right upper and middle lobe (Fig. 3a). Lung (Fig. 4a) and thyroid biopsies demonstrated CD1a and S-100 positivity, confirming the diagnosis of LCH. She was diagnosed with PLCH accompanied by thyroid involvement. Induction chemotherapy (AraC, vincristine [VCR], and prednisolone [PSL]) according to the protocol JLSG-02, described by the Japan Langerhans Cell Histiocytosis Study Group [8], was immediately initiated and continued with chest tube drainage for repeated bilateral PTX.

**Fig. 5:**
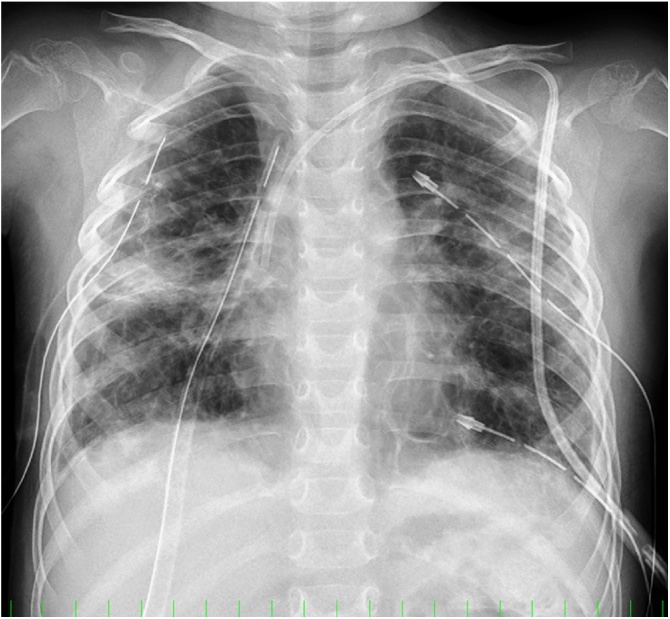
Chest X-ray showing multinumber chest tubes due to repeated bilateral PTX.

However, her pulmonary lesions progressed, and the number of bullae gradually increased (Fig. 2b-c). Suddenly, she experienced chest pain and developed tachycardia and hypotension. CT showed extensive bullae in the right lobe, which exerted a mass effect on the surrounding lung parenchyma, causing a left mediastinal shift (Fig. 2d). Tension bullae developed in the right lung 1 month after initiating treatment. Emergency thoracoscopy-assisted resection of the bullae and ligation of pulmonary cysts to prevent the occurrence of PTX were performed (Fig. 3b). Although she was doing well immediately after the operation, on the tenth postoperative day, she developed a tension bulla in the left lung (Fig. 2e). Emergency thoracotomy with plication of the bulla was performed (Fig. 3c). A second course of a different chemotherapy (doxorubicin [ADR], VCR, cyclophosphamide [CPM], PSL, and cyclosporine A [CyA]) was administered [8]. On the tenth postoperative day, the patient again developed dyspnea and tachycardia. Chest X-ray and CT suggested return of the right bulla and mediastinal shift (Fig. 2f), and reoperation was performed. During the operation, no evidence of exacerbation of the bulla was detected, but a right tension PTX was identified. Repeated lung biopsy showed no sign of active LCH (Fig. 4b). In our case, intermittent positive pressure ventilation (IPPV) could not be avoided to maintain the oxygen saturations during all operations because of technical problems. Although her condition improved after the third operation, subsequent repeated PTXs occurred and were difficult to treat. She required multinumber procedures and multinumber chest tubes. (Fig. 5). Eventually, she underwent autologous blood pleurodesis. Although she experienced no additional episodes of symptomatic PTX and tension bullae, there was evidence of some residual PTX on a follow-up CT scan (Fig. 2g). Follow-up neck CT showed resolution of thyroid swelling. The TSH level was 3.62 μIU/ml with normal T3 and T4 levels. She was discharged home after four months.

She had one episode of PTX one month after discharge. Currently, she is receiving maintenance therapy as an outpatient and her respiratory status is stable, and has no PTX or other complications. During the 12-month follow-up, the pulmonary lesions also showed a tendency for improvement (Fig. 2h). She requires careful ongoing follow-up.

## 3 Discussion

This case illustrates two important points: 1) although it is difficult to treat recurrent PTX and tension bullae in advanced PLCH, continuous treatment of the primary disease (LCH) and the complications of pulmonary lesions can improve prognosis; and 2) positive-pressure ventilation should be performed with extreme caution in suspected PLCH.

PLCH can present alone or together with other disorders to form a multisystem disease. Pulmonary involvement is present in 10% of all patients with LCH and 23–50% of children with multisystem LCH with a mean age of 11.9 months [[1], [2], [3]]. Disease progression is accompanied by the appearance of small cysts and bullae with subsequent fibrosis and honeycombing. Spontaneous PTX occurs in approximately 10% of children with PLCH and can be a fatal complication [2,3]. Spontaneous PTX likely recurs due to bursting of thin-walled subpleural cysts [9]. There are currently no published recommendations for the management of PTX associated with pediatric PLCH. Underwater-sealed drainage and pleurodesis can be used to manage patients with refractory recurrent PTX. Lung transplantation is an option for patients with advanced, progressive PLCH like our case. However, it is not uncommon to be asked whether to perform a pleurodesis in these patients who may come to lung transplantation in the future. Therefore, we did not perform any pleurodesis in the first surgical approaches. Meanwhile, tension bullae is a serious disease associated with critical respiratory and circulatory perturbations arising from raised intrathoracic pressure following fast and uncontrolled growth of a cyst.

Patients with PTX and tension bullae due to PLCH tend not to respond to conventional treatment due to underlying fibrotic lungs, which are fragile and tend to collapse easily. A worsening of these symptoms in a large number of cases indicates the need for immediate surgical treatment. However, the small number of cases has made it difficult to establish a consensus on the timing and importance of treatment using pleurodesis and thoracotomy. Surgeons must therefore choose the best surgical management method on a case-by-case basis.

Fortunately, our patient showed good response to treatment and was without reactivation for 1 year. Although lung disease was traditionally considered a poor prognostic indicator in LCH, recent studies indicate that it does not adversely affect the outcome. Most children with PLCH have a good prognosis and most lung lesions improve or stabilize [10]. Therefore, although it is sometimes difficult to treat recurrent PTX and tension bullae in advanced PLCH, continuous treatment of the primary disease (LCH) and the complications of pulmonary lesions can improve prognosis.

In our patient, contralateral pulmonary lesions were exacerbated following surgeries conducted under general anesthesia. When anesthetizing infants with lung lesions, an important consideration is whether positive-pressure ventilation will cause cardiopulmonary deterioration [11]. Yule et al. reported three children who developed recurrent intrathoracic air leaks [12]. They suggested that general anesthesia be used with caution in children with active LCH because IPPV may cause bullae formation with a sudden deterioration in lung function. Anesthesia for infants with pulmonary interstitial emphysema in LCH remains a challenge due to a risk of possible cardiopulmonary deterioration [13]. It is generally believed that positive pressure ventilation is contraindicated before the chest is open and spontaneous ventilation should be retained during induction and maintenance of anesthesia. But it is clinically very difficult and may be harmfull. In our case, we attempted selective bronchial intubation and unilateral positive ventilation of the opposite lung but failed. Therefore, positive pressure ventilation could not be avoided to maintain the oxygen saturation.

The patient underwent three surgeries. After the first surgery, intraoperative positive-pressure ventilation may have produced tension bullae in the opposite lung, causing compression of the lung and mediastinum. This required an emergency second operation on the opposite thorax. Moreover, on the tenth day after the second operation, the patient again developed tension PTX. As in our case, repeated PTX and tension bullae may be a postoperative complication in children with LCH. It is likely that IPPV during the last operation caused additional lung bullae formation, which subsequently ruptured and/or facilitated the development of PTX on the right side. Indeed, repeated lung biopsy showed no sign of active LCH.

One factor contributing to this outcome is that positive-pressure ventilation during general anesthesia in patients with pulmonary lesions with severe emphysematous changes, like in our patient, can cause damage to the lung, destruction of the lesions, tension PTX, and tension bullae. Therefore, positive-pressure ventilation should be performed with extreme caution in suspected PLCH.

## 4 Conclusion

Treatment of PLCH accompanied by recurrent pneumothorax and tension bullae is challenging. The condition is addressed by treating the primary disease in parallel with combined modality treatment, including surgical operation for complications.

## Conflicts of interest

The authors declare no conflicts of interests or disclosures.

## Sources of funding

This work received no funding.

## Ethical approval

This study is exempt from ethical approval in our institution.

## Consent

Written informed consent was obtained from the patient’s parents for the publication of this case report. A copy of the written consent is available for review by the Editor-In-Chief of this journal, upon request.

## Author’s contribution

SY made the conception and design of this case report. Authors other than SY contributed to the collection, analysis, and interpretation of the data. SY wrote the draft manuscript, and other authors performed the critical revision of the manuscript. All authors gave final approval of the version to be published. SY has overall responsibility and guarantees the scientific integrity.

## Registration of research studies

Not required.

## Guarantor

Satoshi Yokoyama, Tatsuo Nakaoka, Daisuke Fukao, Koji Yokoyama,

Shigeto Hara, Keigo Hamahata.

## Provenance and peer review

Not commissioned, externally peer-reviewed
